# Ocular Wnt/β-Catenin Pathway Inhibitor XAV939-Loaded Liposomes for Treating Alkali-Burned Corneal Wound and Neovascularization

**DOI:** 10.3389/fbioe.2021.753879

**Published:** 2021-10-26

**Authors:** Yueyang Zhong, Kai Wang, Yin Zhang, Qichuan Yin, Su Li, Jiaming Wang, Xiaobo Zhang, Haijie Han, Ke Yao

**Affiliations:** ^1^ Eye Center, The Second Affiliated Hospital, School of Medicine, Zhejiang University, Hangzhou, China; ^2^ Zhejiang Provincial Key Lab of Ophthalmology, The Second Affiliated Hospital, School of Medicine, Zhejiang University, Hangzhou, China; ^3^ The First Affiliated Hospital, School of Public Health, Institute of Translational Medicine, State Key Laboratory of Experimental Hematology, School of Medicine, Zhejiang University, Hangzhou, China

**Keywords:** liposomes, Wnt/β-catenin pathway, XAV939, corneal wound, corneal neovascularization

## Abstract

Corneal wound involves a series of complex and coordinated physiological processes, leading to persistent epithelial defects and opacification. An obstacle in the treatment of ocular diseases is poor drug delivery and maintenance. In this study, we constructed a Wnt/β-catenin pathway inhibitor, XAV939-loaded liposome (XAV939 NPs), and revealed its anti-inflammatory and antiangiogenic effects. The XAV939 NPs possessed excellent biocompatibility in corneal epithelial cells and mouse corneas. *In vitro* corneal wound healing assays demonstrated their antiangiogenic effect, and LPS-induced expressions of pro-inflammatory genes of IL-1β, IL-6, and IL-17α were significantly suppressed by XAV939 NPs. In addition, the XAV939 NPs significantly ameliorated alkali-burned corneas with slight corneal opacity, reduced neovascularization, and faster recovery, which were attributed to the decreased gene expressions of angiogenic and inflammatory cytokines. The findings supported the potential of XAV939 NPs in ameliorating corneal wound and suppressing neovascularization, providing evidence for their clinical application in ocular vascular diseases.

## Introduction

As one of the most vulnerable parts of the eye that is exposed to the external environment, the cornea is consistently susceptible to potential infectious and traumatic damages (Mobaraki et al., 2019; Chen F. et al., 2020; Han et al., 2020a). In addition, the constantly increasing number of refractive surgeries has prompted corneal wound healing an important clinical problem. Corneal wound involves a series of complex and dynamic pathological processes, including cell death, inflammatory and immune response, corneal neovascularization (CNV), and limbal stem cell deficiency, which compromise corneal transparency and lead to decreased vision (Roshandel et al., 2018; Ziaei et al., 2018). Previous studies have illustrated the effectiveness of inhibiting inflammatory responses and blocking angiogenic signaling pathways in accelerating corneal wound healing, providing potential therapeutic options that target multiple processes in treating corneal wounds (Ellenberg et al., 2010; Chandler et al., 2019; Yang J. et al., 2020, Yang et al., 2020 S.; Rebibo et al., 2021).

The evolutionarily highly conserved Wnt signaling pathway regulates various processes, including proliferation, polarization, migration, apoptosis, and stem cell maintenance and differentiation, which are related to systemic development and various congenital and developmental diseases (Reya and Clevers, 2005; Klaus and Birchmeier, 2008; Clevers and Nusse, 2012; Feng et al., 2020). In addition, previous studies have implicated the indispensable role of the Wnt pathway in developmental and pathological ocular angiogenesis (Ouyang et al., 2014; Wang Z. et al., 2019). In the canonical Wnt signaling pathway, Wnt ligands bind to the receptor complex of frizzled receptors and low-density lipoprotein receptor–related protein 5/6, which results in the stabilization of β-catenin and the degradation of Axin (Huang et al., 2009; Clevers and Nusse, 2012). Without phosphorylation, β-catenin is translocated into the nucleus and binds to the transcription factors to activate Wnt target genes (Huang et al., 2009; Clevers and Nusse, 2012). Consequently, various inhibitors along the Wnt signaling pathway have been identified to regulate abnormal gene activation, among which XAV939, a small molecule that stabilizes Axin and stimulates β-catenin degradation, exhibits therapeutic promise in treating Wnt pathway–dependent diseases (Huang et al., 2009). Further studies have identified the anti-inflammatory and antitumor activities of XAV939, yet its potential in treating ocular vascular diseases has not been reported (Li et al., 2018; Almasoud et al., 2020; Zhang et al., 2020).

One obstacle to the treatment of ocular diseases is poor drug delivery and maintenance (Fan et al., 2021; Zhang et al., 2021). The topical eye drop solution, which is one of the most common and preferred methods of drug administration to the eye, is readily excreted through tear drainage and peribulbar blood flow. For instance, it is estimated that 60% of the drug is eliminated within 2 min and is completely discharged within 15 min, resulting in low drug absorption (Jumelle et al., 2020). Moreover, eye drops are unlikely to deliver drugs with insoluble properties. As such, the liposome delivery system, one of the first polymeric nanoparticles approved by the FDA, allows the delivery of hydrophobic and hydrophilic compounds with varying sizes and properties (Kompella et al., 2013; Anselmo and Mitragotri, 2019; Chen X. et al., 2020; Chang et al., 2021; Shi et al., 2021). As biodegradable and biocompatible lipid assemblies, liposomes can achieve sustained drug release to the ocular surface and high penetration into the inner structure (Kompella et al., 2013; Peng et al., 2020; Tavakoli et al., 2020; Yuba, 2020). Various studies have reported prolonged release, improved efficacy, and reduced toxicity of liposome-capsulated drugs, thereby providing substantial potentials for clinical applications (Eriksen et al., 2018; Jin et al., 2019; Ma et al., 2020).

In this study, we constructed a Wnt/β-catenin pathway inhibitor, XAV939-loaded liposomes (XAV939 NPs), and investigated its anti-inflammatory and antiangiogenic effects. The effects of corneal wound healing and CNV suppression were investigated in an alkali-burned mouse model to explore its therapeutic potential in treating ocular vascular diseases.

## Materials and Methods

### Preparation of XAV939 NPs

The liposomes loaded with XAV939 were prepared using a thin-film hydration method as described in previous studies (Alfaifi et al., 2020; Ye et al., 2020). In brief, 3 mg of XAV939 (MedChemExpress, China), 8 mg of L-α-phosphatidylcholine (soybean lecithin, J&K Scientific, China), 3 mg of 1,2-distearoyl-sn-glycero-3-phosphoethanolamine-N-[methoxy (polyethylene glycol)-2000] [DSPE-PEG (2000), AVT Pharmaceutical Technology Co., Ltd, China], and 2 mg of cholesterol (J&K Scientific, China) were dissolved in 10.0 ml chloroform in a round-bottom flask. The mixture was evaporated under reduced pressure in a rotary evaporator for 3 h, and a homogeneous phospholipid film was formed. Then, 10 ml of double-distilled water was added into the round-bottom flask to hydrate the phospholipid film for 1 h, and the liposomes were ultrasonicated for 1 min to form a uniform suspension. The final concentration of XAV939 in the liposomes was 1 mM.

### Characterization of XAV939 NPs

The hydrodynamic size and polydispersity index of the XAV939 NPs were measured by dynamic light scattering (DLS, Zetasizer Nano ZS90, Malvern, United Kingdom). The samples were filtered through a 0.22-μm Millipore filter before taking the measurements, and their morphologies were characterized by cryo-electron microscopy (Cryo-EM) on a 200 kV Talos F200C microscope (FEI, Holland).

### Cell Culture

Human corneal epithelial cells (HCEs) were purchased from the American Type Culture Collection (United States). Human umbilical vein endothelial cells (HUVECs) were purchased from Beyotime Biotechnology (China). The HCEs were cultured with DMEM/F12 (Corning, United States) with 10% fetal bovine serum (FBS) and 1% penicillin–streptomycin. The HUVECs were cultured with DMEM (Corning, United States) with 10% FBS and 1% penicillin–streptomycin. All cells were incubated in a 37°C/5% CO_2_ humidified chamber.

### Cytotoxicity Assays

#### Cell Counting Kit-8 Assay

Cytotoxicity was measured with a cell counting kit-8 (CCK-8) assay (Dojindo, Japan) (Han et al., 2017; Han et al., 2020b). Briefly, the HCE cells were seeded in a 96-well plate to reach the density of 1 × 10^4^ per well. The cells were incubated with a 100 μl DMEM/F12 medium and treated with 10% phosphate buffered saline (PBS) or XAV939 NPs of different XAV939 equivalent concentrations: 0, 0.1, 1, 10, 25, and 50 μM. After 24 h of incubation, 10 μl CCK-8 solution was added to each well and maintained for 2 h at 37°C according to the manufacturer’s instructions. The optical density at 450 nm (OD_450nm_) was measured using an absorbance microplate reader (Bio-rad iMark, United States).

#### Calcein-AM/Propidium Iodide Assay

For Calcein-AM/propidium iodide (PI) assay (Yeasen, China), the HCE cells were seeded in a 12-well plate with a density of 1 × 10^5^ cells per well. The cells were incubated with 1 ml DMEM/F12 medium and treated with 10% PBS or XAV939 NPs of different XAV939 equivalent concentrations: 0, 0.1, 1, 10, 25, and 50 μM. After 24 h of incubation, the medium was removed, and the cells were rinsed with PBS three times. Subsequently, the cells were treated with 0.67 μM Calcein AM and 1.5 μM PI and were incubated for 15 min at 37°C. The cells were then observed under a fluorescence microscope (Leica, Germany), and images of live (green) and dead (red) cells were obtained.

### Quantitative Reverse Transcription-Polymerase Chain Reaction

The HCEs were seeded in a 12-well plate until they reached a density of 1 × 10^5^ cells per well. The cells were first incubated with a fresh DMEM/F12 medium containing 1% FBS, and 10% PBS, XAV939 (10 μM), or XAV939 NPs (10 μM XAV939 equivalent) for 24 h. To induce an inflammatory response, the cells were treated with 1 μg/ml lipopolysaccharides (LPS, Sigma Aldrich, United States) for 2 h. The total RNA of the HCEs was extracted using TRIzol reagent (Invitrogen, Carlsbad, United States) and reverse-transcribed using the PrimeScript™ RT Master Mix (Takara Bio Inc., Japan). Reverse transcription–polymerase chain reaction (RT-PCR) was performed using the ChamQTM SYBR Color qPCR Master Mix (Vazyme Biotech Co., Ltd., China) on a 7500 Fast Real-Time PCR System (Thermo Scientific, United States). The relative gene expressions of inflammatory cytokines, including interleukin (IL)-1β, IL-6, and IL-17α, were analyzed and normalized to the GAPDH level. The relative quantitation was calculated with the comparative cycle threshold Ct (*∆∆Ct*) method.

### Cell Migration Assay

Vascular endothelial cells proliferation has been validated as an initial step in angiogenesis, followed by migration, adhesion, and differentiation (Lamalice et al., 2007). Therefore, for the cell migration assay, we adopted HUVECs, which have been abundantly used (Lamalice et al., 2007; Wang M. et al., 2019). These cells were seeded in a 6-well plate until they reached a density of 2 × 10^5^ cells per well. After a confluent monolayer was formed, a wound was generated in each well by a sterilized 200 μl peptide tip, and the cells were washed three times with PBS. The cells were then treated with 1 μg/ml recombinant human vascular endothelial growth factor-165 (rhVEGF_165_, PeproTech Inc., United States) and combined with 10% PBS, XAV939 (10 μM) or XAV939 NPs (10 μM XAV939 equivalent) and incubated with a fresh DMEM medium containing 1% FBS. Cell migration and wound closure were observed at 0, 12, and 24 h with an Olympus inverted light microscope, and microphotographs were taken at 10× magnification with an Oplenic digital camera. The scratch widths and the percentage of wound closure were quantified using ImageJ software (National Institutes of Health, Bethesda, United States).

### Immunoblotting

The HUVECs were seeded in a 6-well plate with a density of 2 × 10^5^ cells per well. These cells were then treated with 1 μg/ml rhVEGF_165_ and combined with the previously indicated treatments. After 24 h of incubation, the cells were rinsed three times with PBS. The cell lysates were harvested with a lysis buffer with protease and phosphatase inhibitors (Sangon Biotech, China). After lysing on ice for 30 min, the cell lysates were centrifuged at 14,000 × g for 15 min at 4°C. The protein concentration was calculated using a bicinchoninic acid protein assay kit (Thermo Scientific, United States) and normalized. Thirty micrograms of protein were resolved with 6–10% sodium dodecyl sulfate-polyacrylamide gel electrophoresis and electro-transferred to polyvinylidene fluoride membranes. The membranes were then blocked with a protein-free rapid blocking buffer (Epizyme, China) and immunoblotted with the following antibodies: rabbit monoclonal anti–β-catenin (Cell Signaling Technology, United States, 1:1000), rabbit monoclonal anti-Axin 1 (Cell Signaling Technology, United States, 1:1000), and rabbit monoclonal anti–β-actin (Cell Signaling Technology, United States, 1:1000). All quantitative data were normalized to β-actin as an endogenous control. ImageJ software was used to analyze the band intensity.

### Animals

Female C57BL/6 mice aged 6–8 weeks were purchased from Shanghai SLAC Laboratory Animal Co., Ltd, China. All animal experiments strictly abided by the Association for Research in Vision and Ophthalmology Statement for the Use of Animals in Ophthalmic and Vision Research and the guidelines of Zhejiang University Administration on Laboratory Animal Care.

### *In Vivo* Biocompatibility Assessment

To assess the biocompatibility of the liposomes, 10 μl of XAV939 NPs (10 μM XAV939 equivalent) was topically administered to the eyes of the healthy mice in the experimental group. The control group received 10 μl of saline solution. On days 3, 7, and 14, the mice were anesthetized, and the ocular surface was observed with a slit lamp microscope. After 14 days of treatment, the mice were sacrificed, and the eyeballs and major visceral organs, including the heart, liver, spleen, lung, and kidney, were fixed to perform hematoxylin and eosin (H&E) staining for histological observation.

### Alkali-Burned Injury Mouse Model

The alkali-burned injury mouse model was developed to generate corneal wound and CNV, as previously described (Anderson et al., 2014). A mouse was first anesthetized through a peritoneal injection of sodium pentobarbital (100 mg/kg) and topical administration of 0.5% proparacaine solution to the corneal surface of the right eye. Then, a 2-mm-diameter filter paper was soaked in 1 N sodium hydroxide (NaOH) solution and tapped on a dry filter paper for 5 s to absorb the excess alkali. The NaOH-soaked filter paper was placed on the center of the mouse’s right cornea for 30 s and then rinsed with 10 ml of PBS for 60 s. All alkali-burned injury procedures were performed by the same investigators to minimize variability, and the mice were randomly divided into three groups: saline, XAV939 (10 μM, dissolved in 0.1% v/v DMSO/saline), and XAV939 NPs (10 μM XAV939 equivalent). All alkali-burned eyes were treated with 10 μl of the abovementioned eye drop solution twice daily for 14 consecutive days.

### Clinical Examination

On days 3, 7, and 14, all mice were weighed and observed with a slit lamp microscope, and representative images were taken. Two examiners independently performed clinical evaluations and reported a final score according to the established methods (Anderson et al., 2014):

1) Corneal opacity (0–4): 0 = completely clear; 1 = slightly hazy, iris and pupil easily visible; 2 = slightly opaque, iris and pupil detectable; 3 = opaque, pupils hardly detectable; and 4 = completely opaque, pupil undetectable.
2) CNV (0–3): 0 = no CNV, 1 = CNV at the corneal limbus, 2 = CNV spanning over the corneal limbus to the corneal center, and 3 = CNV invading the corneal center.
3) Neovessels (0–3): 0 = no neovessels; 1 = neovessels detectable under a surgical microscope; 2 = neovessels easily visible under a surgical microscope; and 3 = neovessels visible to the naked eye.

CNV was calculated based on the range of clock hours of corneal neovessels. The CNV size was calculated as follows:

$$S=\frac{C}{12}*3.1416*\left[r^{2}-{\left(r-l\right)}^{2}\right],$$

where S = CNV size, C = clock hours of CNV, r = radius of the mouse cornea, and l = length of the longest neovessel from the limbus.

### Immunohistochemistry

After 14 days of treatment, the mice were sacrificed, and the right eyes were enucleated under a stereomicroscope (Leica, Germany), followed by fixation with 4% paraformaldehyde (PFA, Biosharp, China) overnight. The eyeballs were then embedded in paraffin, sectioned, and stained with H&E. The sections were then observed under a microscope (Leica, Germany), and representative photographs were taken. The central corneal thickness was measured and analyzed independently by two examiners using ImageJ software. To assess the biosafety and toxicity of XAV939 and XAV939 NPs, the alkali-burned mice were sacrificed and major visceral organs were enucleated to perform H&E staining.

### Immunofluorescence

On day 15, the mice were sacrificed, and the eyeballs were harvested for corneal flat mount staining. After being fixed in 4% PFA overnight, the corneas with the limbal area were carefully dissected under a stereomicroscope. To make the cornea lie flat, four incisions were made from the periphery toward the center cornea. After being rinsed with PBS with Tween-20 (PBST) three times, the corneas were blocked with 1% bovine serum albumin, 2% goat serum, and 0.2% Triton X-100 in PBS for 2 h and incubated with rabbit anti–mouse CD31 antibody (Abcam, United Kingdom, 1:50) prepared in a blocking buffer at 4°C overnight. The corneas were then rinsed with PBST six times and incubated with Alexa-Fluor 555-conjugated goat anti-rabbit secondary antibody (Invitrogen Life Technologies, United States, 1:1000) for 2 h at room temperature, followed by rinsing with PBST six times. Immunofluorescent images of the corneas were obtained using a fluorescence microscope. The neovessels length and the ratio between the CNV area and whole cornea area were calculated with ImageJ software.

### qRT-PCR

After 14 days of treatment, the mice were sacrificed, and the right corneas were excised. The total RNA of the corneas was extracted using TRIzol reagent. Two corneas were pooled as one biological replica, and three samples were conducted for each group. The methods used for qRT-PCR have been described in the previous section. The relative gene expression quantification included the following: *Vegfs*, *Vegfrs*, *Mmps*, *α-Sma*, *Cd31*, and *Il-6*. The gene expressions were normalized to the GAPDH level, and the relative quantitation was calculated with the *∆∆Ct* method.

### Statistical Analyses

All statistical analyses were performed using SPSS 26.0 (IBM Corp., United States). The data were presented as the mean with its standard error of mean (SEM). Comparisons among three or more groups were performed for statistical significance using one-way analysis of variance (ANOVA). The statistical significance was defined as *p* < 0.05.

## Results and Discussion

### Preparation and Characterization of XAV939 NPs

**FIGURE 1:**
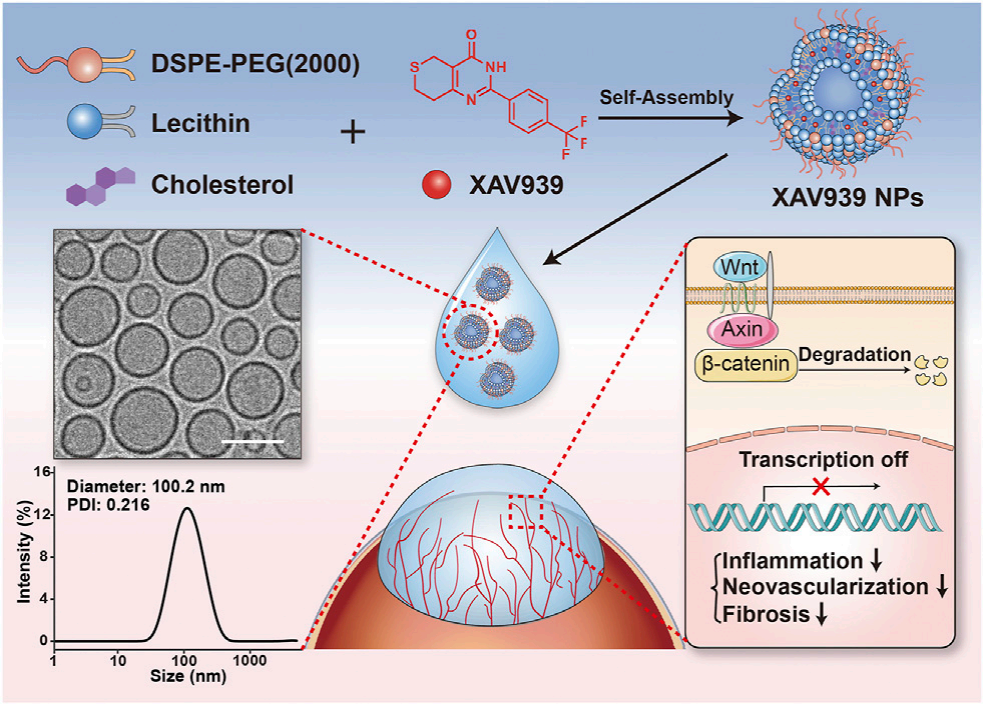
Schematic diagram depicting the XAV939 NPs with corneal wound healing and anti-CNV effect.

As illustrated in Figure 1, the hydrophobic drug XAV939 was loaded into liposomes formed of L-α-phosphatidylcholine, DSPE-PEG (2000), and cholesterol to obtain biocompatible XAV939 NPs. The encapsulation efficiency of the XAV939 NPs was calculated to be 91.2%. The XAV939 NPs exhibited a low polydispersity index (PDI = 0.216) with a hydrodynamic diameter of approximately 100.2 nm using DLS measurements (Figure 1). The cryo-EM images show that XAV939 NPs are spherical and well-dispersed round-shaped vesicles and are predominantly unilamellar (Figure 1), similar to the previously reported liposomes (Cong et al., 2021).

### *In Vitro* Cytotoxicity Assessment

**FIGURE 2:**
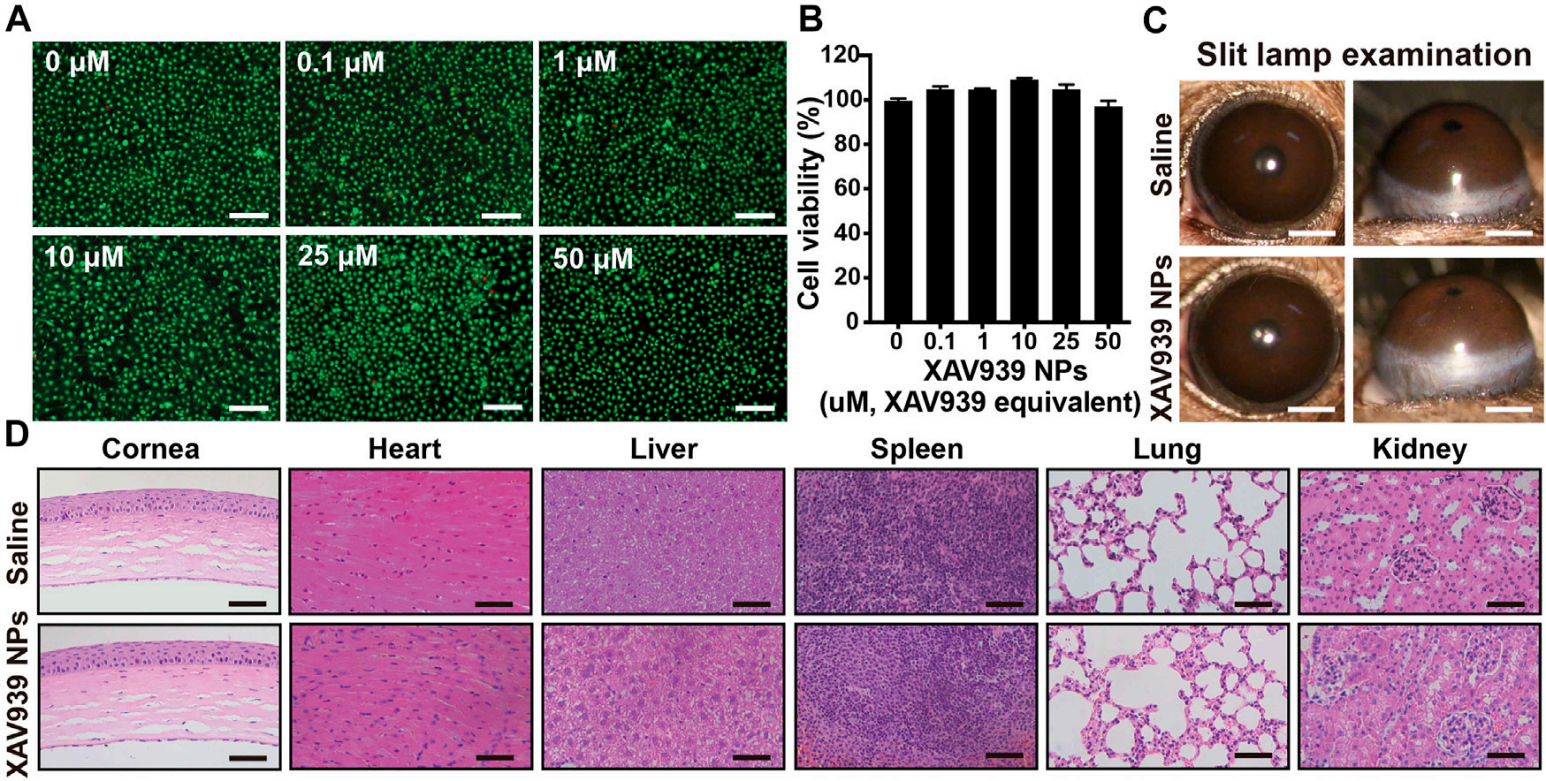
*In vitro* and *in vivo* biocompatibility assessments. Calcein-AM/PI assay **(A)** and CCK-8 assay **(B)** of HCEs following exposure of different concentrations of XAV939 NPs. Results are presented as the mean ± SEM. *n* = 5. Scale bar: 200 μm. **(C)** Representative images of slit lamp examination following 14 days treatment of saline and XAV939 NPs (10 μM XAV939 equivalent). Scale bar: 1 mm. **(D)** Representative images of H&E staining of the cornea, heart, liver, spleen, lung, and kidney following 14 days treatment of saline and XAV939 NPs (10 μM XAV939 equivalent) in healthy mice. Scale bar: 100 μm.

To assess the cytotoxicity of XAV939 NPs, HCE cells incubated with different concentrations of XAV939 NPs were evaluated by the CCK-8 assay and Calcein-AM/PI assay. After incubation for 24 h, cytotoxicity was assessed with the Calcein-AM/PI assay, where green fluorescence represents live cells and red fluorescence represents dead cells. As shown in Figure 2A, few red cells were observed over the concentrations ranging from 0.1 to 50 μM. The results were confirmed by the CCK-8 assay, which indicated the relatively low cytotoxicity of XAV939 NPs with equivalent drug concentrations (Figure 2B). The cell viability treated with XAV939 NPs over a range of concentrations was not significantly different. Even at the highest equivalent concentration of 50 μM, the cell viability treated with XAV939 NPs was 96.9%. Taken together, the *in vitro* cytotoxicity assays confirmed good cytocompatibility of the XAV939 NPs, and the equivalent concentration of 10 μM XAV939 was chosen for further experiments.

### *In Vivo* Biocompatibility Assessment in Healthy Mice

The *in vivo* biocompatibility evaluation of XAV939 NPs was assessed with a corneal stimulation test conducted on healthy mice eyes. As shown in Figure 2C, topical XAV939 NPs (10 μM XAV939 equivalent) or saline were administered to healthy mouse eyes twice daily and observed consecutively for 14 days. Clinical examination exhibited no visible signs of corneal opacity, inflammation, conjunctival congestion, CNV, or hemalopia in the mice’s eyes in both groups. After 14 days of examination, the mice were sacrificed to conduct corneal and systemic anatomical morphology assessments by H&E staining. Anatomical segments of the cornea and major visceral organs of the XAV939 NPs group showed a comparable morphology to the control group (Figure 2D). No signs of inflammatory infiltration, cellular lesions, or acute or chronic physiological toxicity were observed in either group, indicating good biocompatibility of XAV939 NPs *in vivo*. Previous studies have reported the *in vivo* biocompatibility of 10 µM of XAV939 in mouse models of dermal fibrosis and cutaneous and cartilage injury (Distler et al., 2013; Bastakoty et al., 2015). Based on these preliminary results, it can be hypothesized that XAV939 NPs embrace satisfactory ocular tolerance and can act as secure theranostic agents for further ocular drug delivery applications.

### *In Vitro* Antiangiogenesis Effect

**FIGURE 3:**
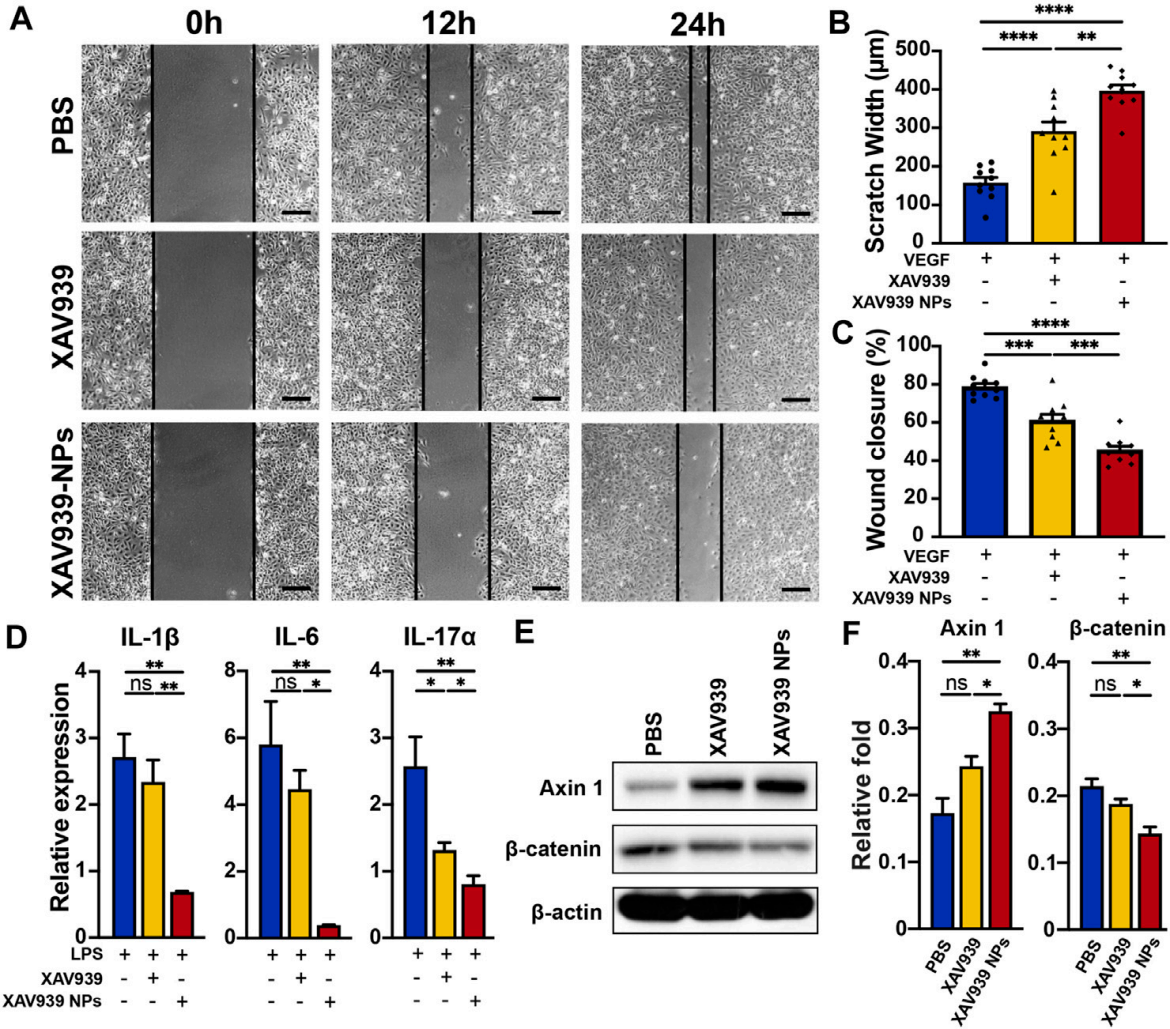
*In vitro* antiangiogenesis and anti-inflammatory effects through Wnt/β-catenin signaling pathway regulation. **(A)** Scratch wound migration assay of HUVECs stimulated with rhVEGF_165_ (1 μg/ml) following exposure of PBS, XAV939 (10 μM), and XAV939 NPs (10 μM XAV939 equivalent). Scale bar: 200 μm. Quantification of the scratch widths **(B)** and the percentage of wound closure **(C)**. Results are presented as the mean ± SEM. *n* = 10. **(D)** Relative expression of mRNA levels of IL-1β, IL-6, and IL-17α of HCEs stimulated with LPS (1 μg/ml) following exposure of PBS, XAV939 (10 μM), and XAV939 NPs (10 μM XAV939 equivalent). Results are presented as the mean ± SEM. *n* = 3. Western blot analysis **(E)** and quantification of the relative protein levels **(F)** of Wnt pathway components Axin 1 and β-catenin following pretreatment of PBS, XAV939 (10 μM), and XAV939 NPs (10 μM XAV939 equivalent). Results are presented as the mean ± SEM. *n* = 3. (One-way ANOVA, *p < 0.05, **p < 0.01, ***p < 0.001, ****p < 0.0001, ns, not significant).

Previous studies have verified the vital roles of the vascular endothelium in wound healing and angiogenesis, which are indicated by excessive cellular proliferation and migration (Wilson et al., 2001; Dorrell et al., 2007). Here, the antiangiogenesis effect of XAV939 NPs (10 μM XAV939 equivalent) was assessed with a HUVEC cell migration assay. The average scratch width and percentage of wound closure observed after 24 h of indicated incubation at each time point are presented in Figure 3A. The average initial scratch wound of the HUVECs was 736 μm in each group. Following induction by 1 μg/ml rhVEGF_165_, significantly increased proliferation and migration of the HUVECs were observed in the control group, with 320 μm scratch width at 12 h and 157 μm scratch width at 24 h (Figure 3B). Nevertheless, pretreatment with XAV939 and XAV939 NPs remarkably attenuated the stimulation of rhVEGF_165_. Previous evidence has also suggested that XAV939 significantly inhibited the proliferation and migration of vascular smooth muscle cells after stimulation with platelet-derived growth factor-BB, which is attributable to the regulatory role of XAV939 in the Wnt signaling pathway (Chen et al., 2016). In particular, HUVECs pretreated with XAV939 NPs presented significantly wider scratch wounds (396 vs. 291 μm, *p* < 0.01) and delayed wound closure (45 vs. 61%, *p* < 0.001) than the XAV939 group, suggesting an enhanced antimigration effect of XAV939 NPs (Figures 3B,C). This is attributable to the high affinity and similarity of the liposomes delivery system with the cell membrane, which in turn facilitates an effective internalization and cellular uptake of the nanoparticles (Skotland et al., 2017; Patel et al., 2020).

### *In Vitro* Anti-Inflammatory Effect

As one of the initial pathological processes of corneal injury, inflammation can be ascribed as a precursor to the development and deterioration of CNV (Lee et al., 2014; Huang et al., 2017). To evaluate the potential anti-inflammatory effect of XAV939 NPs, HCEs were stimulated with 1 μg/ml of LPS for 2 h, and the expressions of the pro-inflammatory genes, including IL-1β, IL-6, and IL-17α, were examined. A significantly upregulated pro-inflammatory gene expression was found in the control group. For instance, the relative expression of IL-6 achieved more than a five-fold elevation after LPS stimulation. However, cells pretreated with 10 μM of XAV939 and XAV939 NPs exhibited significantly suppressed pro-inflammatory gene expressions (Figure 3D). The XAV939 NPs group showed the lowest levels of IL-1β (*p* < 0.01 vs. control and *p* < 0.01 vs. XAV939), IL-6 (*p* < 0.01 vs. control and *p* < 0.05 vs. XAV939), and IL-17α (*p* < 0.01 vs. control and *p* < 0.05 vs. XAV939). Our results are in accord with those obtained in a previous study that identified the anti-inflammatory activity of XAV939 in both human bronchial epithelial cells and HUVECs (Jang et al., 2019). During the inflammatory process, the Wnt signaling pathway was activated, and XAV939 was found to dose-dependently suppress LPS-induced pro-inflammatory signaling (Koopmans et al., 2017). Therefore, with the liposome delivery system, XAV939 NPs mediated substantial and sustained drug release and exhibited a greater anti-inflammatory effect.

### *In Vitro* Wnt/β-Catenin Signaling Pathway Regulation

The Wnt/β-catenin signaling pathway controls various biological processes, and abnormal activation of the Wnt pathway has been observed in various ocular congenital and developmental vascular diseases (Huang et al., 2009; Chen et al., 2011; Lee et al., 2012; Wang et al., 2015). Previous studies have identified that XAV939 can stimulate β-catenin degradation by stabilizing Axin and selectively inhibiting abnormal β-catenin–mediated transcription (Huang et al., 2009). To evaluate the mechanism of XAV939 NPs and the modulation of the Wnt/β-catenin signaling pathway, the protein levels of the key modulators of the pathway were investigated (Figure 3E). The pretreatment of 10 μM of XAV939 significantly increased the protein levels of Axin 1 and that of XAV939 NPs exhibited the effect to a greater extent (*p* < 0.01 vs. control and *p* < 0.05 vs. XAV939). Additionally, XAV939 and XAV939 NPs decreased the abundance of β-catenin (*p* < 0.01 vs. control and *p* < 0.05 vs. XAV939), which is the key regulator of the transcription of Wnt pathway-responsive genes (Figure 3F). Our results showed that XAV939 displayed a significant inhibitory effect on the abnormally activated Wnt signaling pathway, and the XAV939 NPs group exhibited better performance. Consistent with previous evidence, the inhibitors of the Wnt signaling pathway exhibit therapeutic potential for ocular neovascularization diseases (Chen et al., 2007; Hu et al., 2013).

### *In Vivo* Anti-CNV and Corneal Wound Healing Effects

**FIGURE 4:**
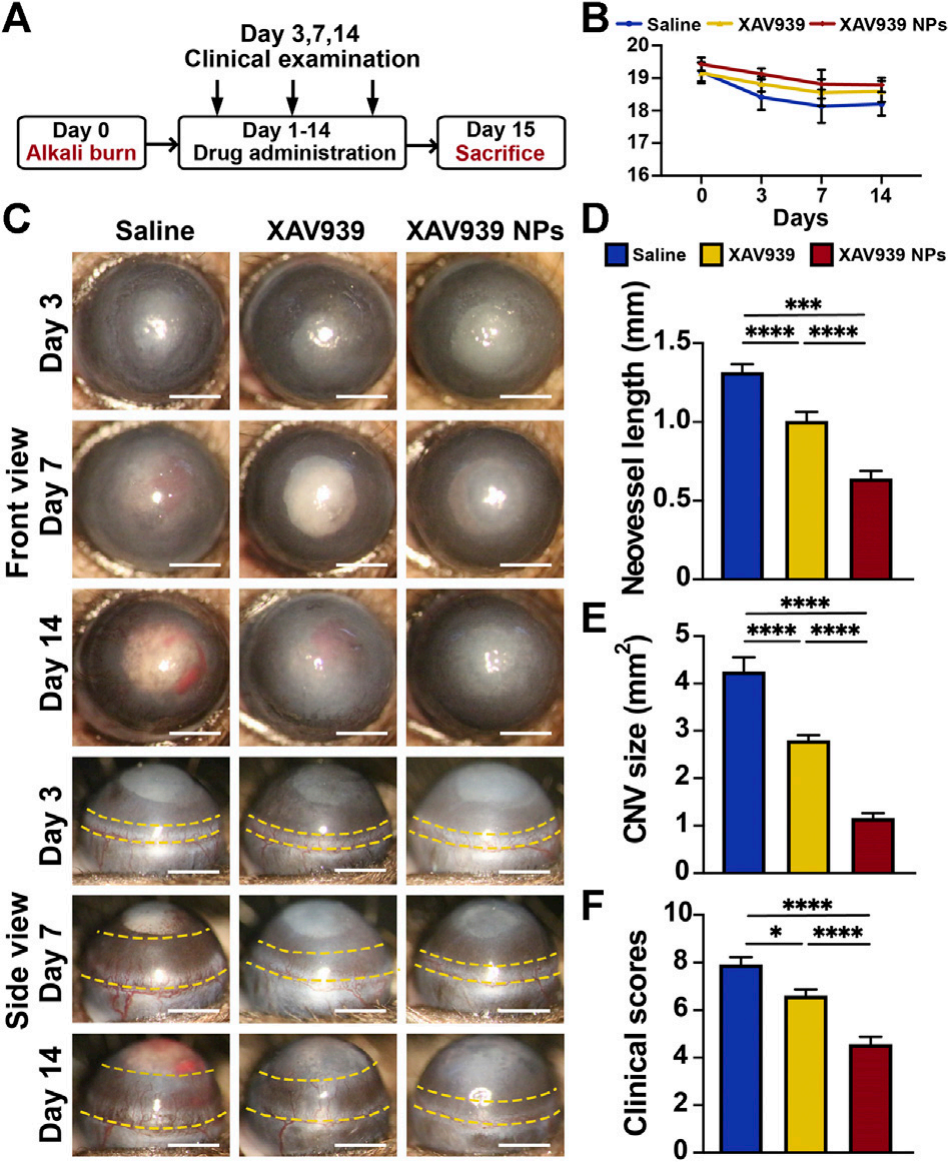
*In vivo* anti-CNV and corneal wound healing effects. **(A)** Schematic timeline of the experimental process. **(B)** Representative front and side images of slit lamp examination on day 3, day 7, and day 14 of the indicated treatments. Yellow dot lines indicate the range of CNV. Scale bar: 1 mm. **(C)** Mice’s weights during the clinical observation of 14 days treatment. Results are presented as the mean ± SEM. *n* = 6. Quantification of the neovessel length **(D)**, CNV size **(E),** and clinical scores **(F)** on day 14 with indicated treatments. Results are presented as the mean ± SEM. *n* = 6. (One-way ANOVA, *p < 0.05, ***p < 0.001, ****p < 0.0001).

As one of the most severe causes of corneal wound, alkali-burned corneas suffer from immediate epithelial defects, followed by opacification, CNV, and fibrosis in the later stage (Kubota et al., 2011). Based on the above findings, the efficacy of XAV939 NPs in promoting corneal wound healing and inhibiting CNV was further assessed using an alkali-burned injury mouse model. Briefly, alkali-burned mice were topically administered with the indicated treatments twice daily for 14 days, and clinical examination was conducted with a slit lamp microscope, after which the mice were sacrificed for histological assessments (Figure 4A). During the 14 days of observation, the body weights of the mice in each group were steady and comparable (Figure 4B). Figure 4C presents the representative front and side photographs of morphological differences induced by different treatments. Generally, corneal opacity and edema were observed on day 3 after the alkali burn, reached their peak on day 7, and gradually weakened afterward in all treatment groups. After the injury, the corneas underwent rapid and dynamic regeneration and repairment (Chandler et al., 2019). Specifically, mice treated with XAV939 NPs had significantly reduced opacification and edema compared to that of the XAV939 group and the saline group during the observation, suggesting an ameliorated inflammatory response.

With respect to CNV, the neovessels were found to expand over the limbal vascular plexus on day 7 and reached the peak on day 14 in all groups. The mice treated with saline presented with the most apparent neovessels, which expanded over the corneal limbus on day 7 and gradually invaded the corneal center. During the corneal healing process, mice treated with XAV939 NPs consistently showed slower development and growth of neovessels than the XAV939 group and the saline group. Notably, a slight intraocular lens opacity was observed in all treatment groups, which indicated the development of cataract. According to previous studies, such phenomena are attributable to repeated anesthetization, the stimulus of strong light from the slit lamp, low body temperature, and eye dryness after an alkali burn (Ridder et al., 2002; Lee et al., 2020). Therefore, avoiding unnecessary anesthetics use and light exposure, keeping the ocular surface moisturized, and maintaining body temperature may prevent the formation of lens opacity.

Additional quantifications of CNV size, neovessel length, and clinical scores were calculated and compared among the groups on day 14 (Figures 4D–F). In the control group, the CNV size was 4.25 mm^2^ with a neovessel length of 1.32 mm. The XAV939 group had an average CNV size of 2.81 mm^2^ and a neovessel length of 1.03 mm. Mice treated with XAV939 NPs presented with a significantly smaller CNV size of 1.25 mm^2^ and neovessel length of 0.66 mm (*p* < 0.001 for the control group; *p* < 0.001 for the XAV939 group). Furthermore, the evaluation of the clinical scores indicated significantly better corneal wound healing in the XAV939 NPs group (4.57 points) than in the XAV939 group (6.60 points, *p* < 0.001) and the control group (7.91 points, *p* < 0.001). As expected, XAV939 NPs treatment had better suppression of the ocular inflammatory response and CNV than XAV939 alone. It is speculated that hydrophobic XAV939 was relatively insoluble in the mice’s eyes and was therefore readily cleared away, which may hamper the treatment effect. On the other hand, the XAV939 NPs with a liposomal delivery system achieved better bioavailability and longer retention on the ocular surface, thereby providing better therapeutic efficacy. With further consideration on the price, consecutive administration of the XAV939 NPs (10 μM XAV939 equivalent) for 14 consecutive days is estimated to cost $ 0.86, which is fairly affordable compared to $ 1.3 of 0.1% sodium hyaluronate eye drops.

### Histological and Immunofluorescent Analyses *In Vivo*

**FIGURE 5:**
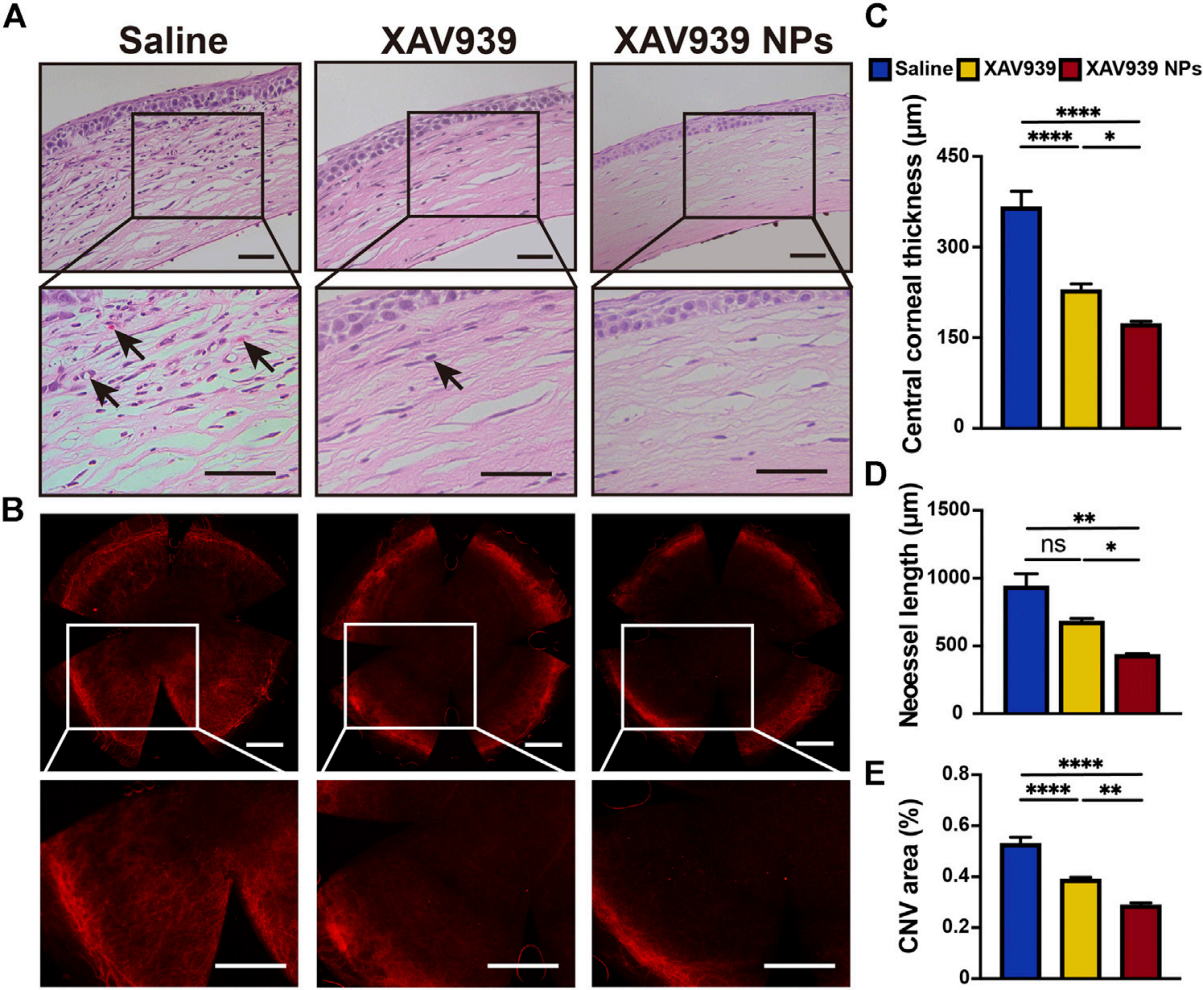
Immunohistochemistry and immunofluorescence assessments of the corneal sections. **(A)** H&E staining of the corneal sections following 14 days of the indicated treatment. The black arrows mark the CNV sites. Scale bar: 50 μm. **(B)** CD31 corneal flat mount staining of the corneal sections following 14 days of the indicated treatment. Scale bar: 500 μm. Quantification of the central corneal thickness **(C)**, neovessel length **(D)**, and CNV area (%) **(E)**. Results are presented as the mean ± SEM. *n* = 3. (One-way ANOVA, *p < 0.05, **p < 0.01, ****p < 0.0001, ns, not significant).

Histological morphology of healthy and alkali-burned mice eyes in all treatment groups was observed with H&E staining (Figure 5A). After the alkali burn, the corneas became generally loosened and frangible. Consistent with the clinical examination, the control group was marked with severely destroyed and exfoliated laminal squamous epithelium, edematous stromal layers, disordered collagen fibers, and more CNV. In addition, more inflammatory infiltration, edema, and epithelial layer detachment were observed. The average central corneal thickness in the control group was 368.3 μm, compared to the values of 231.1 μm noted for the XAV939 group (*p* < 0.001) and 174.5 μm for the XAV939 NPs group (*p* < 0.001) (Figure 5C). The corneal thickness was thinner and more regularly arranged in the XAV939 and XAV939 NPs groups. In the XAV939 group, better morphology and less edema were found, but still presented with slight CNV. In contrast, owing to prolonged retention on the cornea surface, XAV939 NPs-treated corneas showed apparent repairment and inhibition of CNV compared to the other groups.

To better visualize the CNV of the cornea in all groups, corneal flat mount immunofluorescent staining of the vascular endothelial cell marker CD31 was conducted. The CD31 molecule not only facilitated the formation of intercellular junctions between endothelial cells but also signaled the leukocytes to the vasculature (Mansouri et al., 2006). As displayed in Figure 5B, excessive growth of neovessels was found to expand to the cornea center in the saline group. Significant suppression of CNV was observed in the XAV939 and XAV939 NPs groups. CNV areas and neovessel lengths were quantified and compared between the groups. In the saline group, the neovessels expanded over half of the whole corneas with an area percentage of 54% and a length of 924.5 μm (Figures 5D,E). CNV in mice treated with XAV939 was slightly ameliorated, with the CNV size observed to be 38% (*p* < 0.0001 vs. saline group) and a neovessel length of 680.7 μm (ns. vs. saline). The XAV939 NPs group almost recovered from injury, with a CNV area of 29% (*p* < 0.0001 vs. saline; *p* < 0.01 vs. XAV939) and a length of 435.5 μm (*p* < 0.01 vs. saline; *p* < 0.05 vs. XAV939). Therefore, the corneal wound healing and therapeutic efficacy of XAV939 NPs might be related to the downregulation of CD31. Taken together, these results indicate that the XAV939 NPs demonstrated a significant advantage over free XAV939 in attenuating corneal wound and CNV after alkali burns.

### Angiogenic and Inflammatory Gene Expression Assessment

**FIGURE 6:**
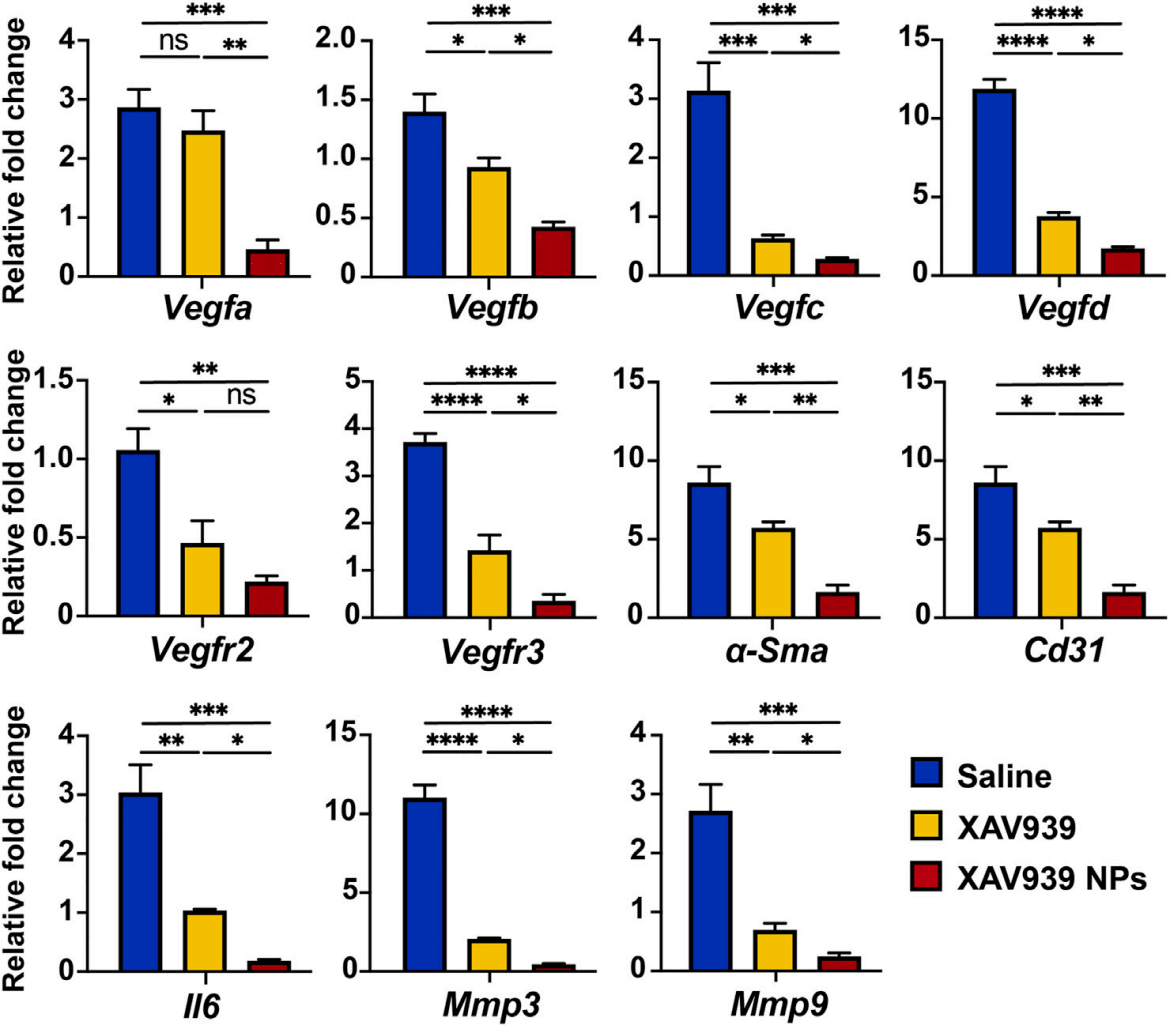
Relative gene expression analyses of *Vegfa, Vegfb, Vegfc, Vegfd, Vegfr2, Vegfr3, α-Sma, Cd31, IL-6, Mmp3,* and *Mmp9* for each group following 14 days of the indicated treatment. Results are presented as the mean ± SEM. *n* = 3 (two corneas served as one biological replica). (One-way ANOVA, *p < 0.05, **p < 0.01, ****p < 0.0001, ns, not significant).

Alkali-burn injury to the cornea involves a complex and dynamic inflammatory and immune response which consequently leads to inevitable CNV and fibrosis in the later stage (Sakimoto et al., 2012; Huang et al., 2017; Sun et al., 2018). In the angiogenesis cascade, VEGFs and their receptors have been identified as the main critical regulating factors (Ellenberg et al., 2010; Apte et al., 2019). According to previous evidence, the Wnt signaling pathway is an upstream pathway regulating VEGFs, and its inhibitors have displayed satisfactory efficacy in inhibiting retinal neovascularization formation (Lee et al., 2012; Hu et al., 2013; Yu et al., 2019). To assess the potential effect of XAV939 NPs on the pathogenesis of CNV, the relative gene expression levels of mice corneas were measured for angiogenic factors (Figure 6). In our study, the relative expressions of angiogenic genes were significantly reduced following the XAV939 NPs treatment. The XAV939 NPs group displayed significantly lower levels of *Vegfa* (*p* < 0.001 vs. saline; *p* < 0.01 vs. XAV939), *Vegfb* (*p* < 0.001 vs. saline; *p* < 0.05 vs. XAV939), *Vegfc* (*p* < 0.001 vs. saline; *p* < 0.05 vs. XAV939), and *Vegfd* (*p* < 0.0001 vs. saline; *p* < 0.05 vs. XAV939). Significantly decreased expressions of *Vegfr2* and *Vegfr3* were also found in the XAV939 NPs group. Treatment with XAV939 also significantly reduced the elevation of *Vegfs* and *Vegfrs* compared to that of the saline group, but was not comparable to the effect of XAV939 NPs.

During the process of corneal wound healing and CNV, the keratocytes in the corneal stroma begin with the transition to fibroblast and myofibroblast to repair the corneal wound edge, which indicates corneal scarring and fibrosis (Maltseva et al., 2001; Ziaei et al., 2018). Therefore, the relative gene expressions of *α-Sma*, a typical marker for myofibroblasts, and *Cd31*, a typical marker for endothelial cells, were further analyzed. As shown in Figure 6, *α-Sma* was significantly suppressed by XAV939 NPs treatment (*p* < 0.001 vs. saline; *p* < 0.01 vs. XAV939). The expression of *Cd31* was also inhibited in the XAV939 NPs group (*p* < 0.001 vs. saline; *p* < 0.01 vs. XAV939). The results also implied inhibited fibrosis and CNV in mice treated with XAV939 NPs.

During the progress of chemical burn–induced corneal injury, inflammatory signaling has been identified as an initial response. Previous studies have deduced the vital role of *Il-6* trans-signaling in ocular surface inflammation after the alkali burn (Sakimoto et al., 2012; Han et al., 2020c). In addition, overexpressed inflammation-associated target genes, such as Mmps, have also been reported, and the inhibition of inflammatory signaling pathways could alleviate the corneal wound and accelerate the healing process (Vandooren et al., 2013; Lee et al., 2014). Further analyses were conducted for inflammatory-related genes. Up to 10-fold upregulated expressions of *Il-6*, *Mmp3*, and *Mmp9* were observed for the control group. Mice treated with XAV939 and XAV939 NPs showed suppressed expressions of the pro-inflammatory genes. However, better efficacy was observed for the XAV939 NPs group, with inhibited genes of *Il-6* (*p* < 0.001 vs. saline; *p* < 0.05 vs. XAV939), *Mmp3* (*p* < 0.0001 vs. saline; *p* < 0.05 vs. XAV939), and *Mmp9* (*p* < 0.001 vs. saline; *p* < 0.05 vs. XAV939). Altogether, these results reconfirm the therapeutic effect of XAV939 NPs through the regulation of angiogenic, inflammatory-related, and fibrotic-related gene expressions.

### Biosafety Assessments of Alkali-Burned Mice

**FIGURE 7:**
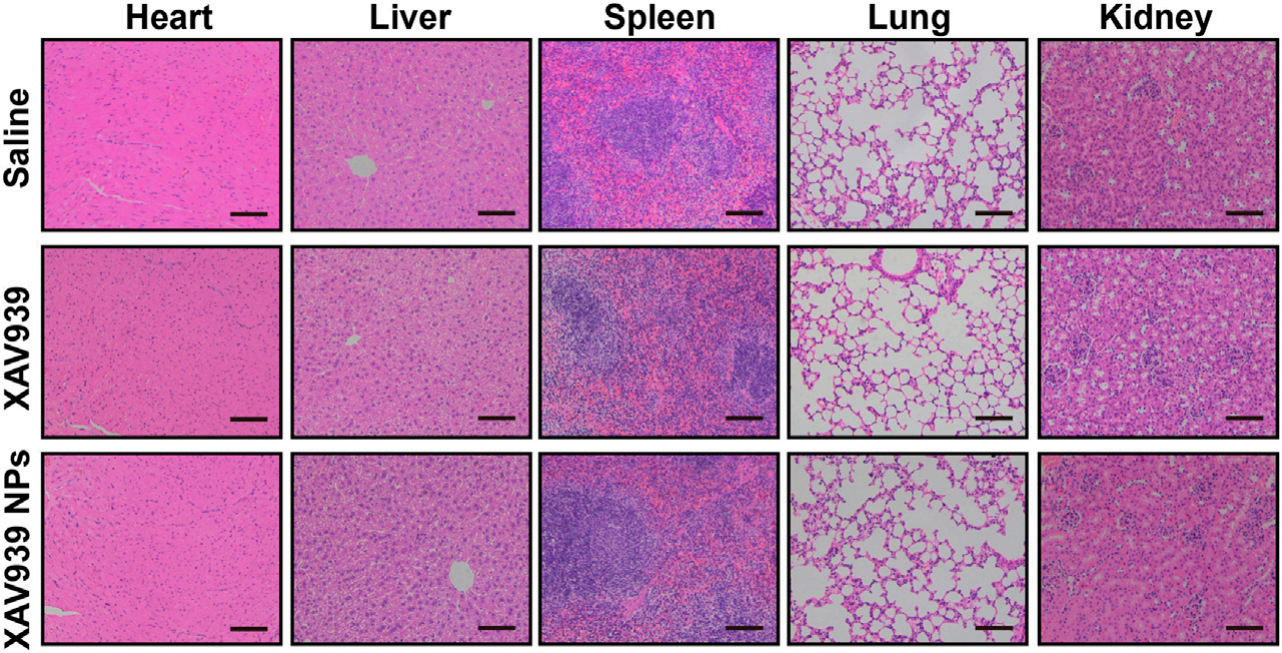
Main visceral organs biosafety assessments in alkali-burned mice. Representative images of H&E staining of the heart, liver, spleen, lung, and kidney following 14 days treatment of saline, 10 μM XAV939, and XAV939 NPs (10 μM XAV939 equivalent). Scale bar: 200 μm.

Further biosafety assessments were conducted to investigate the toxicity of XAV939 and XAV939 NPs by H&E staining of major visceral organs, including the heart, liver, spleen, lung, and kidney, in the alkali-burned mice. As presented in Figure 7, treatment with XAV939 and XAV939 NPs did not exhibit significant morphological or histological differences from the control group. The results indicated the reliable systemic biosafety of topical treatment of XAV939 and XAV939 NPs, thus further proving the superiority and biocompatibility of XAV939 NPs in treating alkali-burned corneal wounds.

## Conclusion

In this study, the therapeutic potential of the Wnt/β-catenin pathway inhibitor XAV939 in treating ocular vascular disease was reported for the first time. In addition, biocompatible liposomes were used to enhance the water solubility and bioavailability of hydrophobic XAV939, enabling ocular application. *In vitro* studies indicated XAV939 NPs’ anti-inflammatory and antiangiogenic efficacy through the regulation of the Wnt signaling pathway, which exhibited an advantage over XAV939 treatment. When topically administered to alkali-burned corneas, XAV939 NPs demonstrated enhanced corneal wound healing and suppressed CNV, and the expressions of angiogenic and inflammatory-related genes were inhibited by XAV939 NPs, further confirming their anti-inflammatory and antiangiogenic effects. Taken together, our results illustrate the therapeutic potential of the ocular liposomes of XAV939, thus providing a promising alternative therapy for corneal wound repair and CNV inhibition.

## Data Availability

The raw data supporting the conclusions of this article will be made available by the authors, without undue reservation, to any qualified researcher.
